# Analysis of XFEL serial diffraction data from individual crystalline fibrils

**DOI:** 10.1107/S2052252517014324

**Published:** 2017-10-20

**Authors:** David H. Wojtas, Kartik Ayyer, Mengning Liang, Estelle Mossou, Filippo Romoli, Carolin Seuring, Kenneth R. Beyerlein, Richard J. Bean, Andrew J. Morgan, Dominik Oberthuer, Holger Fleckenstein, Michael Heymann, Cornelius Gati, Oleksandr Yefanov, Miriam Barthelmess, Eirini Ornithopoulou, Lorenzo Galli, P. Lourdu Xavier, Wai Li Ling, Matthias Frank, Chun Hong Yoon, Thomas A. White, Saša Bajt, Anna Mitraki, Sebastien Boutet, Andrew Aquila, Anton Barty, V. Trevor Forsyth, Henry N. Chapman, Rick P. Millane

**Affiliations:** a Department of Electrical and Computer Engineering, University of Canterbury, Christchurch, New Zealand; b Centre for Free-Electron Laser Science, DESY, Hamburg, Germany; c Linac Coherent Light Source, SLAC National Accelerator Laboratory, Menlo Park, California, USA; d Institut Laue-Langevin, Grenoble, France; e Faculty of Natural Sciences, Keele University, England; f European Synchrotron Radiation Facility, Grenoble, France; g European XFEL GmbH, Hamburg, Germany; h Max Planck Institute of Biochemistry, Martinsried, Germany; i Department of Materials Science and Technology, University of Crete and IESL/FORTH, Crete, Greece; j Max-Planck Institute for the Structure and Dynamics of Matter, Hamburg, Germany; k Université Grenoble Alpes, Grenoble, France; l Lawrence Livermore National Laboratory, Livermore, California, USA; m Photon Science, DESY, Hamburg, Germany; n Department of Physics, University of Hamburg, Hamburg, Germany; p Centre for Ultrafast Imaging, University of Hamburg, Hamburg, Germany

**Keywords:** serial crystallography, coherent X-ray diffractive imaging (CXDI), single particles, molecular orientation determination, crystalline fibrils, amyloid

## Abstract

Serial diffraction data collected at the Linac Coherent Light Source from crystalline amyloid fibrils delivered in a liquid jet show that the fibrils are well oriented in the jet. At low fibril concentrations, diffraction patterns are recorded from single fibrils; these patterns are weak and contain only a few reflections. Methods are developed for determining the orientation of patterns in reciprocal space and merging them in three dimensions. This allows the individual structure amplitudes to be calculated, thus overcoming the limitations of orientation and cylindrical averaging in conventional fibre diffraction analysis. The advantages of this technique should allow structural studies of fibrous systems in biology that are inaccessible using existing techniques.

## Abstract 2

Methods are described for processing XFEL data from individual crystalline fibrils. The methods are applied to data collected at the Linac Coherent Light Source from an amyloid-forming oligopeptide from the adenovirus shaft.

## 1. Introduction   

The development of X-ray free-electron lasers (XFELs) has brought in a new era for the study of biomolecular structures (Chapman *et al.*, 2011 ▸; Fromme, 2015 ▸). In the context of structural biology, the most successful application of XFELs has been through serial femtosecond crystallography (SFX) (Chapman *et al.*, 2011 ▸; Spence *et al.*, 2012 ▸; Chapman, 2015 ▸; Fromme, 2015 ▸; Schlichting, 2015 ▸; Martin-Garcia *et al.*, 2016 ▸). XFELs deliver a coherent femtosecond-duration hard X-ray pulse to a molecular specimen, achieving high-angle elastic scattering before inelastic processes destroy the molecule (Neutze *et al.*, 2000 ▸). The peak brilliance of the X-ray pulses is at least eight orders of magnitude larger than that of synchrotron sources, enabling measurements from nanometre-sized crystals that are too small for use with synchrotron sources (Gati *et al.*, 2017 ▸). Such crystals are easier to obtain than those of at least tens of micrometres across that are required by synchrotron sources. Crystals are usually injected into the XFEL pulse path through a nozzle as a suspension in a liquid jet (Weierstall, 2014 ▸; Nelson *et al.*, 2016 ▸; Oberthuer *et al.*, 2017 ▸) oriented perpendicular to the incident X-ray path, although other sample-delivery methods are also in use (Weierstall *et al.*, 2014 ▸; Chavas *et al.*, 2015 ▸; Sierra *et al.*, 2015 ▸; Roedig *et al.*, 2016 ▸; Martin-Garcia *et al.*, 2016 ▸; Fuller *et al.*, 2017 ▸). The maximum pulse repetition rate at the Linac Coherent Light Source (LCLS) at the SLAC National Accelerator Laboratory is 120 Hz, and diffraction patterns are usually recorded at this rate (Bostedt *et al.*, 2016 ▸). The diffraction frames are pre-processed to select those that result from an X-ray pulse interacting with one or more crystals, as shown by the presence of sharp diffraction peaks, and also to reject frames that are unsuitable due to artifacts, excessive background diffraction *etc*. Auto-indexing is then used to determine the orientation of the crystal in the X-ray beam. The Bragg diffraction intensities are then measured and thousands of the intensity sets are averaged to obtain a set of three-dimensional measured structure amplitudes (Kirian *et al.*, 2010 ▸; White *et al.*, 2012 ▸). Structure determination can then proceed using conventional crystallographic methods such as molecular replacement. Various software packages have been developed for this processing chain (Barty *et al.*, 2014 ▸; White *et al.*, 2012 ▸, 2016 ▸, 2017 ▸; Sauter *et al.*, 2013 ▸; Kabsch, 2014 ▸; Liu & Spence, 2016 ▸). The success of this approach is shown by approximately 100 entries in the PDB of structures determined by SFX (Martin-Garcia *et al.*, 2016 ▸).

A long-term goal of the application of XFELs is imaging of single molecules, or other small particles, without the requirement for crystallization (Neutze *et al.*, 2000 ▸; Aquila *et al.*, 2015 ▸). This is a challenging objective however, because the diffracted intensity is extremely weak in the absence of the coherent amplification provided by a crystal, and difficult to measure above the background diffraction. Fibrous bio­molecular assemblies are long slender systems that are periodic in only their axial direction and thus lie between the crystalline and single-particle cases. The one-dimensional periodicity enhances the diffraction and boosts signal levels above that for general single particles, and these assemblies are thus a potential target for the application of XFELs. XFEL diffraction from such specimens is the subject of this paper.

Some fibrous systems exhibit only one-dimensional crystallinity, *i.e.* they exist as single molecules that are periodic along their long axis, and they give diffraction that is continuous on discrete planes, referred to as layer planes, in reciprocal space. These are sometimes referred to as noncrystalline fibres. Other fibrous systems exhibit some lateral crystallinity in which the molecules pack side-by-side in a regular crystal lattice, and are sometimes referred to as crystalline fibres. These specimens are essentially three-dimensional crystals, but they generally have a large aspect ratio, *i.e.* they are much longer in the axial direction than in the lateral direction, and they are often somewhat flexible. The diffraction can still be thought of as existing on layer planes, but on each layer plane it consists of Bragg reflections, resulting in further enhancement of the diffracted intensity.

In this paper we consider the case of crystalline fibres, which have the advantage that their diffraction is (Bragg) sampled, and thus stronger than for noncrystalline fibres. The weaker and unsampled (or continuous or diffuse) diffraction from noncrystalline fibres is closer to the single-particle case, and its analysis presents additional challenges. In this paper we report on methods we have developed for processing the weak and sparse XFEL diffraction data obtained from single crystalline fibres of an amyloid-forming oligopeptide from the adenovirus shaft. The focus here is on the data processing, and more details of the experiment will be reported elsewhere (Liang, 2018 ▸). Structural interpretation of the data is in process and will be reported in a subsequent publication.

Fibrous, fibrillar or filament assemblies are common in biology and are responsible for a wide variety of biological functions. They include, for example, collagen fibrils, actin filaments, muscle proteins, filamentous viruses, flagella, bacterial pili, microtubules, amyloid fibrils and nucleoproteins. Despite their importance, they are difficult to study at the structural level, in part because their large aspect ratio means that they are reluctant to form three-dimensional crystals. However, X-ray diffraction has played a key role in structural studies of filamentous viruses, microtubules and flagella, as well as more flexible polymeric systems such as polypeptides, polysaccharides and polynucleotides, that can be prepared as stretched fibres (Stubbs, 2001 ▸; Millane, 2010 ▸). The specimens used for these studies, referred to as fibres, consist of a large number of molecules (or molecular assemblies) that are oriented with their long axes approximately parallel. Sometimes they may exhibit some limited crystallinity in a plane normal to their long axes. A large number of molecules are needed in the fibre specimen in order to obtain sufficient diffraction signal from conventional X-ray sources. Importantly, in these specimens the individual molecules (or small crystallites of molecules) adopt different random rotations about the axis of orientation, *i.e.* the specimen is cylindrically disordered. The result of this is that the recorded diffraction intensities are cylindrically averaged about the corresponding axis in reciprocal space. The cylindrical averaging collapses reciprocal space from three dimensions to two, and the diffraction data are collected on a single two-dimensional pattern from a single exposure (since all rotations are represented in the specimen).

The cylindrical averaging of the diffraction substantially reduces its information content compared with the case of a single crystal. Structure determination from such data, referred to as fibre diffraction analysis, is thus more difficult than in conventional single-crystal crystallography, and requires more ancillary information in addition to the diffraction data. The molecules within fibres often adopt helical symmetry and a high order of helical symmetry eases the reconstruction problem, but it is generally still quite underdetermined. Furthermore, alignment of the molecules is not perfect, which produces overlap of the diffraction signals at high resolution, limiting the resolution of the data. This characteristic of fibre specimens is referred to as disorientation, *i.e.* individual molecules are differently oriented relative to the the mean direction of orientation. We will use this term here. A disorientation of no more than a few degrees is generally required for high-resolution structural studies. Disorientation is analogous to mosaicity in single-crystal diffraction, but it is generally more severe in fibre specimens. Other forms of disorder, such as partial crystallinity, can complicate structure determination as they can affect the relationship between the molecular/crystal structure and the diffracted intensities (Stroud & Millane, 1995 ▸, 1996 ▸; Millane, 2010 ▸).

As a result of the difficulties of specimen preparation and the limited amount of diffraction information that is available, the application of fibre diffraction analysis has been quite limited. Considering the number of biologically important such systems, the number of structures that have been determined is small. This represents a significant gap in studies of biomolecular systems. XFELs offer the potential to overcome the primary limitations of traditional fibre diffraction analysis by measuring the diffraction from single molecules, thus opening up diffraction studies of a much wider range of fibrous assemblies than has previously been possible. This results from the possibility of injecting fibrous particles into the X-ray beam using a liquid jet, inducing flow alignment of the individual high-aspect-ratio particles. Aside from providing a novel alignment mechanism, with a low particle concentration, a high-intensity X-ray pulse and a small focus, there is also the possibility of recording diffraction patterns from single individual molecules, *i.e.* as a result of an X-ray pulse intersecting only a single molecule in the interaction volume. Note that this does not require that no X-ray pulses interact with two or more molecules, only that such cases can be detected and removed from the diffraction data set. If such diffraction patterns can be recorded and analysed in such a scenario, then the two major limitations of fibre diffraction analysis are circumvented: orientation and cylindrical averaging. Even if the molecules are imperfectly oriented in the jet, diffraction patterns from *individual* particles suffer no disorientation effect. The only restriction is that orientation in the jet is good enough that there are a sufficient number of molecules oriented close enough to the jet axis that an interpretable diffraction pattern is obtained. This is a weak requirement. Furthermore, diffraction from a single molecule gives data for a particular rotation about its long axis, *i.e.* the individual diffraction patterns are not cylindrically averaged. Different molecules will exist in all different rotations, so that data can be collected for all rotations and, if the individual rotation angles can be determined, the data can be assembled to create a full data set in three-dimensional reciprocal space for a single molecule. The only potential difficulty with the scenario described is signal level. However, coherent enhancement of the diffraction with crystalline fibres, albeit considerably less than for the case of larger three-dimensional crystals, helps to boost the signal level. The main question is whether patterns from single molecules can be identified and oriented. If so, then the signal-to-noise ratio can be improved by averaging, as is done in conventional SFX. The orientation problem is also eased in the case of crystalline fibres that give sampled diffraction.

In order to investigate the potential for single fibre diffraction with XFELs, we conducted experiments with a number of fibrous systems delivered in a liquid jet at the Linac Coherent Light Source (LCLS) (Emma *et al.*, 2010 ▸). Particularly good data were obtained from crystalline fibres of an amyloid-forming oligopeptide from the adenovirus shaft. Adenovirus fibres are trimeric proteins that protrude from the 12 fivefold vertices of the virion. The structure of the fibres has been solved at 2.4 Å resolution by X-ray crystallography, revealing a triple-β spiral (van Raaij *et al.*, 1999 ▸). Isolated peptide sequences from the adenovirus shaft form amyloid-type assemblies (Luckey *et al.*, 2000 ▸), but their detailed structures are unknown. The XFEL data that were collected are from an octapeptide fragment from this system that is known to be a basic self-assembling block. These data were used to develop the processing methods described in this paper.

Amyloid fibrils form when proteins, or fragments of proteins, are converted from the naturally soluble form to insoluble fibrils that can accumulate in a variety of organs (Toyama & Weissman, 2011 ▸). Amyloid and amyloid-type systems are implicated in a wide variety of diseases such as Alzheimer’s disease, Parkinson’s disease, Huntington’s disease, type 2 diabetes mellitus, and a variety of transmissible spongiform encephalopathies (Chiti & Dobson, 2006 ▸). Amyloid fibrils are known to be composed of partially unfolded proteins that self-associate through short segments in a cross-β configuration with the polypeptide chain running perpendicular to the fibre axis. While extensive structural studies have been carried out on many amyloid forms (Kirschner *et al.*, 1986 ▸; Serpell, 2000 ▸; Sunde *et al.*, 1997 ▸; Dobson, 2001 ▸; Jaroniec *et al.*, 2004 ▸; Wille *et al.*, 2009 ▸; Paravastu *et al.*, 2009 ▸; Inouye *et al.*, 2010 ▸), there remain major questions about the amyloid deposits associated with pathologies and the way in which they assemble. High-resolution structures have been obtained by X-ray crystallography of microcrystals of short segments of fibril-forming peptides (Sawaya *et al.*, 2007 ▸). However, the extent to which these structures reflect the native biological fibril is not clear. The quality of fibre diffraction data from typical macroscopic fibre samples as studied using synchrotron sources is usually poor and not suitable for definitive structural analysis, and several rather different models for amyloid fibrils have been proposed (Jahn *et al.*, 2010 ▸). The result is that the underlying motif of amyloid in continuous fibrils is poorly understood. XFELs offer significant opportunities for revealing the structural details of these important biological assemblies, due to the possibility of recording diffraction from single fibrils.

Popp *et al.* (2017 ▸) have recently reported the collection of serial X-ray diffraction data from filament systems at the LCLS using a liquid jet. They observed orientation of the filaments in the jet and were able to perform computational alignment to within 5° in some cases. However, in all cases each diffraction shot was due to at least about 100 individual filaments, and the resolution of the data was no better than 10–20 Å in the best cases. While this study is useful in demonstrating the potential of XFELs for studies of, particularly noncrystalline, filament systems, it did not achieve the high-resolution diffraction from single filaments needed for high-resolution structural studies, which is the objective of the work reported here.

This paper is structured as follows. The experimental setup is briefly described in Section 2 (and will be described in more detail elsewhere). In Section 3, the nature of the diffraction data obtained is described and the basic preprocessing to extract useful patterns is outlined. The various processing steps, including the results, to reduce the data to structure amplitudes are described in Section 4. The implications of the work are summarized in Section 5.

## 2. Experimental   

### 2.1. Sample preparation and pre-characterization   

Amyloid filaments were formed from short peptide sequences related to the adenovirus shaft structure, which in its usual biological context is involved in viral docking to host cells (Philipson *et al.*, 1968 ▸). The structure of this shaft is believed to be a triple-β spiral, as described by van Raaij *et al.* (1999 ▸). Outside this natural context, and in the absence of the registration signal (Papanikolopoulou *et al.*, 2004 ▸) that maintains this structure, the associated peptides form amyloid filaments, as assessed by Congo Red staining, electron microscopy and X-ray fibre diffraction (Luckey *et al.*, 2000 ▸; Papanikolopoulou *et al.*, 2005 ▸).

**Figure 1:**
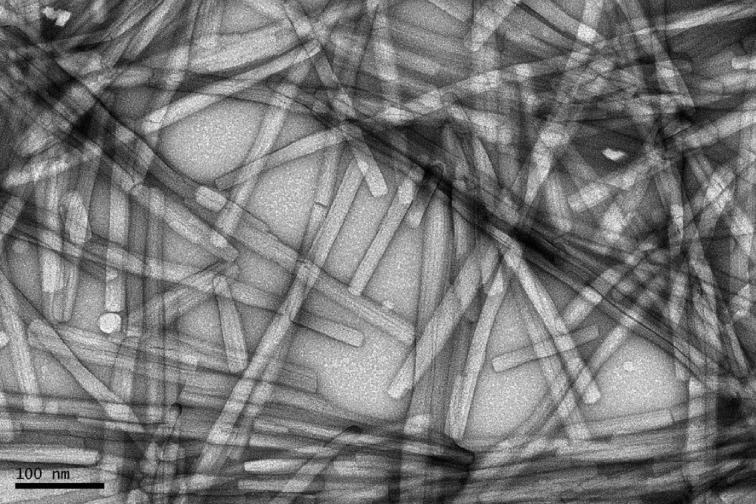
Electron micrograph of the adenovirus amyloid fibrils.

Prior to the experiments at LCLS, samples were characterized by negative-stain electron microscopy (EM), carried out on an FEI T12 microscope at 120 kV with images recorded on an Orius 832 CCD camera. Approximately 4 µl of the sample was applied to the interface of a mica sheet covered with a film of evaporated carbon. The carbon film with the sample adsorbed was then floated off the mica in 2% uranyl acetate and retrieved onto a 400-mesh copper EM grid. The EM images show extended fibrils that are closely monodisperse in width of approximately 200 Å (Fig. 1 ▸). For the XFEL experiments, the peptide material (as a lyophilysed powder) was dissolved in doubly distilled water to a concentration of 10 mg ml^−1^, vortexed, and left to incubate for 24 h at room temperature, during which fibril formation occurred. This stock solution was diluted as required prior to injection into the liquid-jet delivery system.

### 2.2. XFEL experiments   

The experiments were carried out at the Coherent X-ray Imaging (CXI) end station (Liang *et al.*, 2015 ▸) at the Linac Coherent Light Source (LCLS) (Emma *et al.*, 2010 ▸). X-ray pulses with an energy of approximately 6.0 keV (λ = 2.07 Å), a pulse duration of approximately 45 fs and a repetition rate of 120 Hz were focused to a full width at half-maximum (FWHM) of approximately 90 (vertical) × 150 (horizontal) nm. The amyloid sample was diluted to 1.1 mg ml^−1^ and delivered using a tiltable ceramic nozzle, through an inline filter that retained particles larger than 2 µm, as a suspension in a liquid jet of diameter 1.5 µm at a flow rate of about 5 µl min^−1^ (Beyerlein *et al.*, 2015 ▸). The nozzle was tilted at an angle of 10–15° to the normal to the incident XFEL beam to allow access to the region of reciprocal space close to the orientation axis (Millane, 2010 ▸). Diffraction frames were recorded with a Cornell–SLAC Pixel Array Detector (CSPAD) (Hart *et al.*, 2012 ▸) at 120 frames s^−1^ with a sample-to-detector distance of 100 mm. The detector consists of 64 individual panels, each containing pixels spaced by 110 µm, with a grand total of 2.3 M pixels. In total, 2 842 633 frames were recorded (approximately 12 TB of data) during the experiment.

## 3. Preprocessing   

### 3.1. Hit detection   

**Figure 2:**
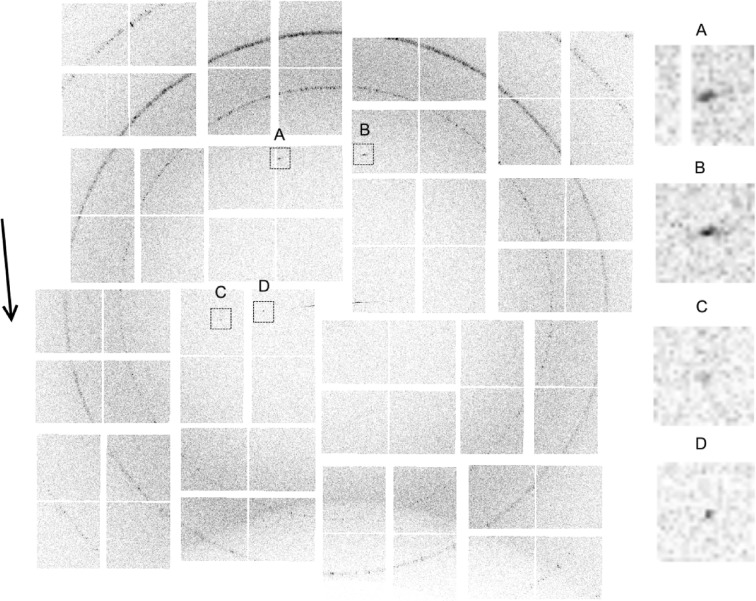
An example single frame, contrast enhanced, showing four diffraction peaks, with detail on the right. The rings are diffraction from the ceramic nozzle. The orientation axis is approximately vertical and the approximate direction of the jet flow is shown by the arrow on the left. Peaks A and B are at ∼4.7 Å and are close to the meridional axis. Peaks C and D are on the equatorial axis and are both to the left of the origin. The ‘jet streak’, *i.e.* diffraction by the water jet, is the small approximately horizontal streak close to the centre of the pattern.

Inspection of individual diffraction frames showed frequent occurrence of a sharp strong reflection at ∼4.7 Å spacing close to the axis of the jet, and sometimes a few sharp peaks on an axis normal to the jet and passing through the origin of the detector. A typical such frame is shown in Fig. 2 ▸. The ∼4.7 Å peak is consistent with typical X-ray diffraction patterns from amyloid fibrils, which show a prominent peak at this spacing close to the filament axis due to stacking of β-sheets along the fibril axis (Toyama & Weissman, 2011 ▸). The proximity of the ∼4.7 Å peaks to the jet axis in many patterns indicates that many fibrils are oriented close to the jet axis, and there is thus good flow alignment of the fibrils in the jet. The sharp equatorial peaks indicate lateral crystallinity of the diffracting fibrils. These features were used as the basis for selecting frames containing useful diffraction data.

Some diffraction frames contain artifacts, including a strong equatorial flare from the water jet, a diffuse diffraction ring from the water, sharp rings from the ceramic injector nozzle, and erroneous peaks due to ice and other parasitic sources, that are not due to the fibrils, and many frames contain no diffraction at all. The XFEL crystallographic data processing software *Cheetah* (Barty *et al.*, 2014 ▸) was used to preprocess the data and select frames containing useful diffraction signals, referred to as ‘hits’, that are suitable for subsequent analysis.

**Figure 3:**
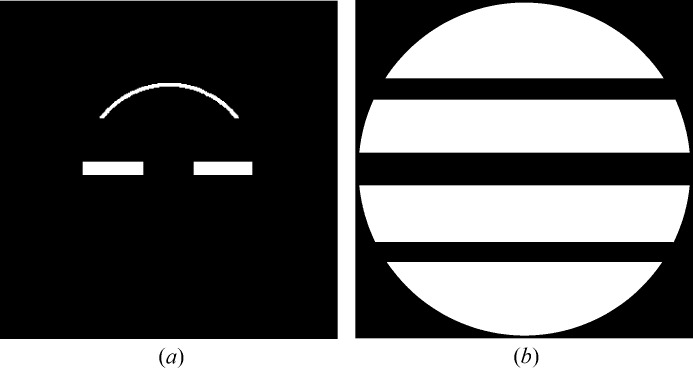
(*a*) The mask used to detect hits and (*b*) the background mask. Note that, as a result of the non-zero tilt of the liquid injector nozzle, the near-meridional peaks generally appear only in the upper half of a diffraction pattern.

Based on the observations described above, a mask is defined that encloses the regions of the near-meridional and equatorial reflections, as shown in Fig. 3 ▸(*a*). Using the hit detection algorithm in *Cheetah*, a hit frame was defined as one that contains at least one peak (within the mask) of more than 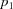 connected pixels, with each pixel exceeding an intensity threshold 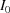. Experimentation showed that the values 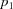 = 5 pixels and 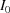 = 30 ADUs (analogue-to-digital units, with ∼20 ADUs per photon at the X-ray energy used here) were suitable for this data set. The pattern shown in Fig. 2 ▸ is a typical hit frame. Using this procedure, 822 969 hit frames were selected from the 2 842 633 original frames, giving a hit rate of 29%. Since, as described below, at least two reflections are needed to orient a diffraction pattern, frames with only one peak are rejected, which gives 372 207 remaining frames. To remove frames containing large strong non-specimen peaks (such as from ice formed on the nozzle, for example), frames with any peak containing more than 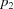 connected pixels are rejected. A value of 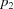 = 40 was found to be suitable, and applying this condition reduced the number of frames to 362 722.

For each hit frame, the number of fibrils that the X-ray pulse intersects is not known *a priori*. Although frames resulting from more than one fibril are theoretically usable, their analysis would be difficult, and we therefore aim to detect and reject such frames. An initial detection of such frames is conducted at this stage, as follows. Since the diffraction pattern is a section through reciprocal space, two reflections from a single fibril cannot be closer than the smallest reciprocal-lattice spacing. Therefore, one diagnostic of diffraction by more than one fibril is the presence of two peaks closer than this minimum distance. Patterns that have any two equatorial peaks closer than this minimum distance (which corresponds to 120 pixels in the case at hand) are rejected. This leaves 43 709 patterns that are used for subsequent analysis. Note that this procedure does not remove all patterns containing diffraction from multiple fibrils, since it is quite possible that the orientations of multiple fibrils are such that they do not produce observed reflections closer than the minimum distance. Further rejection of patterns due to more than one fibril occurs in some of the subsequent steps described below.

### 3.2. Background estimation   

A background function for each frame is calculated as follows. A global background function is first estimated using a background mask that consists of regions where diffraction from the specimen does not occur. The mask is set to the whole of the detector region except for three horizontal bands which include the equator and the upper and lower first layer lines, as shown in Fig. 3 ▸(*b*). The intensity in the frames not recorded as hits is averaged and used as the global background function (Barty *et al.*, 2014 ▸). The background for each frame is calculated as the global background multiplied by a scale factor for that frame which is equal to the ratio of the averaged intensity in the background mask for that frame, divided by the average for the global background. The scale factor accounts for the variability in the background scattering intensity between frames, which is due primarily to fluctuations in the X-ray pulse energy and to positional jitter of the jet which changes the volume of water in the X-ray focus. The background function for each frame is stored for subsequent use.

## 4. Analysis   

### 4.1. Averaged diffraction data   

Each diffraction pattern originates from one, or sometimes a few, fibrils. The individual diffraction patterns, such as that shown in Fig. 2 ▸, are weak, and thus difficult to interpret individually. Over the full set of diffraction patterns, the fibrils exhibit a small spread of orientations in the X-ray beam and the full range of azimuthal orientations about their long axis. Therefore, if the observed patterns are averaged together, we have a pattern averaged over all fibril orientations. This is the equivalent of a conventional fibre diffraction pattern for a specimen with all fibril orientations that are present in the jet.

**Figure 4:**
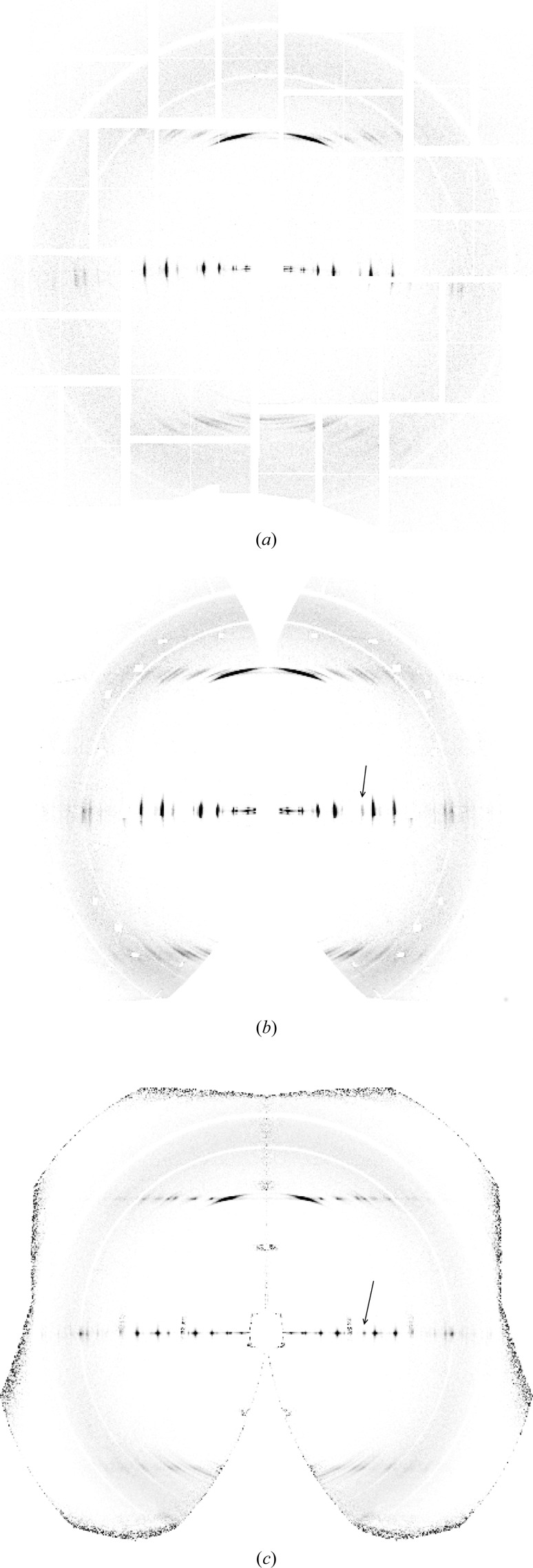
Averaged diffraction patterns (*a*) in detector space and (*b*) mapped into cylindrically averaged reciprocal space, using a tilt of 10° for each pattern. (*c*) Averaged diffraction pattern in reciprocal space after re-orientating each pattern in φ and β as described in the text. The arrows in panels (*b*) and (*c*) show the equatorial reflection used to evaluate the orientation, as described in Section 4.4.

The averaged diffraction pattern is calculated using the 43 709 frames to aid in the initial assessment of the data. The corresponding averaged background is calculated by averaging the individual background functions (derived as described in Section 3.2) for the same frames. The averaged background is subtracted from the averaged diffraction pattern, and the resulting pattern is shown in Fig. 4 ▸(*a*). The averaged pattern resembles a typical crystalline amyloid fibre diffraction pattern, with a strong near-meridional reflection at a spacing of ∼4.7 Å, sharp peaks on the equator and some sharp reflections on the first layer line at an axial spacing of ∼4.7 Å. This confirms that many of the hit frames are due to fairly well oriented amyloid fibril crystallites. Based on the curvature of the equator, the mean fibre tilt is estimated at 10°, and mapping the averaged pattern onto two-dimensional reciprocal space gives the pattern shown in Fig. 4 ▸(*b*).

**Figure 5:**
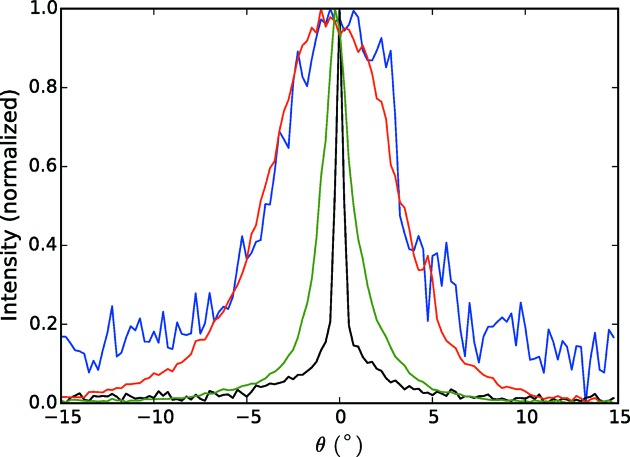
Angular profiles of an equatorial reflection (blue) and the strong reflection on the first layer line (red) in the averaged diffraction pattern shown in Fig. 4 ▸(*b*). Also shown are the angular profiles of the same equatorial (black) and first layer line (green) reflections after reorienting and averaging the diffraction patterns (Fig. 4 ▸ *c*).

The arcing of the reflections in Fig. 4 ▸ is a result of the distribution of orientations of the fibrils, which is due to both variable orientations of the fibrils in the jet and variations in the orientation of the jet. The range of orientations can therefore be estimated from the degree of arcing of the reflections. Angular profiles of an equatorial reflection at reasonably high resolution (shown by the arrow in Fig. 4 ▸ *b*) and of the strong near-meridional reflection on the first layer line are shown by the blue and red curves in Fig. 5 ▸. These indicate a distribution of the fibril orientations with a standard deviation of about 3°. A consideration of the peak broadening and fibril dimensions is described in Appendix *A* [App appa].

In conventional fibre diffraction, Fig. 4 ▸ represents all the diffraction information that is available. However, since the XFEL experiment provides diffraction data from individual fibrils, more information can be obtained. First, by analysis of individual frames, the orientation of the diffracting fibril in the beam can potentially be estimated and the patterns oriented one by one in reciprocal space before averaging. In principle, this removes the effect of disorientation and should extend the resolution of the data. Second, by analysis of individual frames, there is also the potential to determine the rotation of each fibril about its long axis, thereby orientating the pattern in three-dimensional reciprocal space. Averaging these patterns then gives a three-dimensional, as opposed to two-dimensional, diffraction data set. Such an analysis is described in the following.

The important first step is to orient each diffraction pattern in reciprocal space. This corresponds to the usual crystal indexing problem in protein crystallography, which involves determining the individual crystal orientations and the cell constants, and indexing of the reflections on each pattern. Conventional auto-indexing methods in protein crystallography (*e.g.* Powell, 1999 ▸) require a minimum of about 50 reflections, whereas the typical diffraction patterns recorded here contain only two or three reflections. Brewster *et al.* (2015 ▸) describe an indexing method for sparse XFEL patterns with few reflections, but this requires at least five reflections on a pattern.

We address the orientation and indexing problem for the current data set in four steps, which are described in the following subsections. The first step involves determining two angles that define the orientation of the fibril long axis, the second step uses this information to estimate the cell constants, the third step uses the accumulated information to determine the rotation of the fibril about its long axis, and the final step indexes the reflections in three-dimensional reciprocal space.

### 4.2. Diffraction geometry   

**Figure 6:**
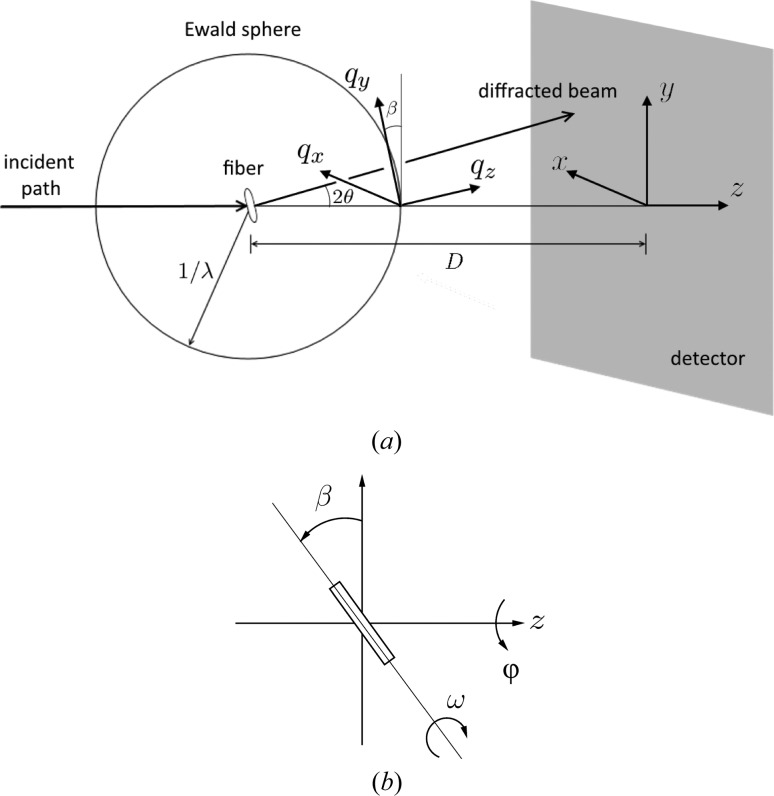
(*a*) Diffraction geometry for 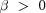, and (*b*) definition of the angles 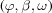 for the orientation of the specimen, looking along the negative *x* axis.

The experiment geometry is shown in Fig. 6 ▸(*a*). We need to consider the orientation of the fibril in the X-ray beam and the mapping from detector space to reciprocal space. Referring to Fig. 6 ▸(*b*), the orientation of the fibre specimen in the beam is defined by the three angles 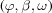. The angle φ denotes rotation of the specimen about the incident X-ray beam and β denotes the tilt of the fibre out of a plane normal to the incident beam. These two angles are equivalent to the ‘rotation’ of the pattern about the incident beam and the ‘tilt’ of the fibre in conventional fibre diffraction (Fraser *et al.*, 1976 ▸; Millane, 2010 ▸). The third angle, ω, denotes rotation of the specimen about its long axis. The angle ω does not appear in conventional fibre diffraction analysis, since in that case molecules or assemblies for all values of ω are present in a single specimen.

Cartesian coordinates on the detector are denoted 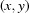, and the X-ray beam travels along the positive *z* axis. Reciprocal-space coordinates are denoted **q** = 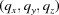, where |**q**| = 2sin(θ)/λ and θ is the Bragg angle. For φ = ω = 0, the 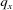 axis is parallel to the *x* axis. The 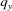 axis is parallel to the fibre axis and, as a result of the fibre tilt, is oriented at an angle β to the *y* axis. For a crystalline specimen, c* is parallel to the 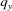 axis.

We consider first the case where φ = β = 0 and then adjust for non-zero angles. In this case, the centre of the Ewald sphere is at **q** = (0, 0, −1/λ). The distance from the Ewald sphere centre to the position 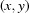 on the detector is
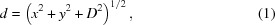
where *D* is the specimen-to-detector distance. The reciprocal-space coordinates corresponding to the position 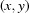 on the detector are then given by
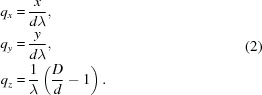



In conventional fibre diffraction, a coordinate system 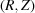 is used in cylindrically averaged reciprocal space, where *Z* is parallel to the long axis of the molecule (Millane, 2010 ▸), so that in terms of the coordinate system 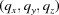,
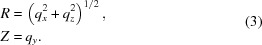
Note that 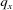 and 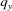 are individually determined from *x* and *y* since we have assumed that ω = 0. If ω is unknown, which is often the case, then only *R*, and not 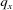 and 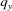, can be determined from *x* and *y*.

The orientation of reciprocal space, and thus its projection onto the detector, for a particular diffraction pattern depends on the fibre orientation 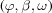. The reciprocal-space co­ordinate system **q** is first transformed through the angles φ and β. The rotation by φ transforms **q** to the rotated frame 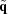 with
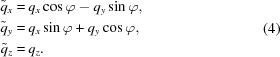
Subsequent rotation by the tilt angle β transforms 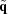 to 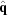 such that 
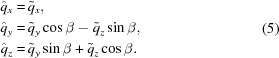
In the rotated coordinate system, equation (3) is replaced by 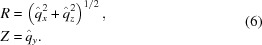



### 4.3. Determination of φ and β   

As described above, the orientation of each fibril is determined by first determining the angles φ and β for each diffraction pattern. This needs to be done for diffraction patterns that contain only a few reflections. Consider two observed reflections with detector coordinates 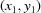 and 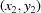, and known reciprocal-space *Z* coordinates 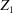 and 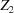, respectively. Referring to equations (5) and (6) then gives
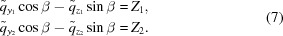
Consider first the case of two equatorial reflections. Substituting from equations (2) and (4) with 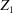 = 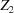 = 0 allows equations (7) to be combined to give
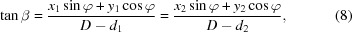
where 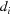 = 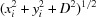. Rearrangement of equation (8) gives
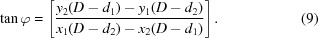
Using the coordinates of the two equatorial reflections, φ can be calculated using equation (9) and then β calculated using equation (8).

For non-equatorial reflections, the calculation of φ and β is more complicated. The *Z* values of the reflections would need to be known and, since the equations do not separate as above, φ and β would need to be jointly determined numerically. Furthermore, there are two solutions for β and the ambiguity is not necessarily easily resolved.

In the amyloid data set, the usable diffraction patterns, extracted as described above, contain at least two equatorial reflections. They also frequently contain the strong reflection on the first layer with a spacing of ∼4.7 Å, on both sides of the meridian. This is a result of the strength of this reflection and the fact that, with the average tilt used, it is frequently close to a diffracting condition.

There are therefore two options for determining φ and β. The first is to use only the equatorial reflections, as this gives a simple direct calculation as described above. The second option is to use four reflections (two equatorial and two first layer line) when available. However, incorporation of first layer line reflections leads to a number of difficulties and uncertainties. The *Z* value of the first layer line reflections would need to be either accurately known or determined jointly with the fibril orientations. The solution would need to be determined by optimization and ambiguities resolved, as described above. Furthermore, because of the strength of the first layer line reflection, it is likely that in many patterns they are actually partials. This means that they appear at slightly variable reciprocal-space coordinates with the correct fibril orientations, and the effect of this on the orientation estimates would need to be assessed. For these reasons we have chosen, at least initially, to use the first option of estimating φ and β from the equatorial reflections only. We are investigating the incorporation of first layer line data into orientation determination and evaluating the issues described above, and the results will be reported in a subsequent paper.

**Figure 7:**
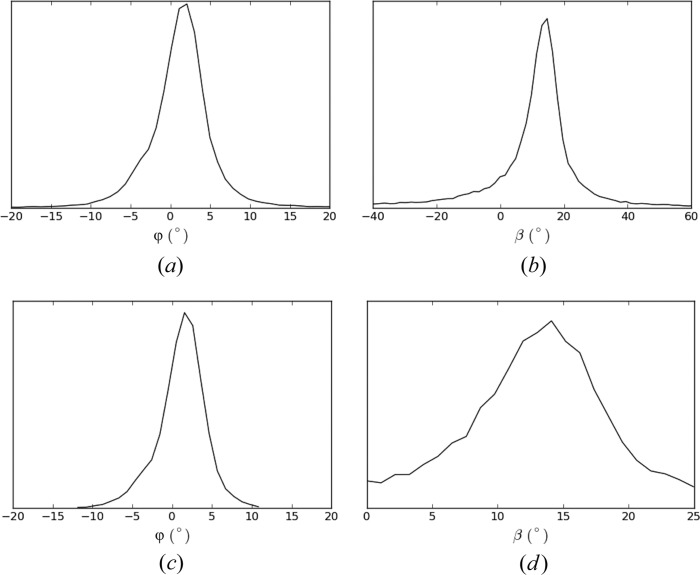
Distributions of φ and β, (*a*) and (*b*) for all accepted patterns, and (*c*) and (*d*) after removing patterns with the estimated β outside the range 0–25°.

The values of φ and β for each of the 43 709 patterns are determined as follows. For each pair of equatorial reflections on a pattern, φ and β are calculated as described above. For patterns with three or more reflections, the resulting set of values are averaged. The distributions of the resulting values of φ and β are shown in Fig. 7 ▸(*a*). Inspection of the figure shows a distribution of φ with an FWHM of 5.6°. The distribution of β has a mean of 10°, an FWHM of 10.0° and quite long tails. For an axisymmetric distribution of fibril orientations (which is expected), the distributions of φ and β should be similar, aside from different means. The broader and long-tailed distribution of the estimated β compared with φ is due to the estimate of β being more sensitive to errors in the reflection positions than is the estimate of φ. The distribution of φ is therefore considered to be a better representation of both distributions. The long-tailed distribution for β indicates that the values in the tails are likely to be in error. Therefore, for each diffraction pattern, any individual calculated β value that is outside the range (0, 25°) is not used in calculating the value of β for that pattern. As a result, some patterns may not be assigned a valid β value, and these patterns are rejected. This leaves 33 788 patterns with assigned φ and β values, and the distributions of φ and β are shown in Fig. 7 ▸(*b*). The FWHMs of φ and β are now 5.2° and 10.6°, respectively, with a mean β of 13°. Although the FWHM values are little changed, this set of patterns is considered to have more reliably determined orientations.

In order to reconcile the differing φ and β distributions, the sensitivity of the determination of φ and β to errors in the reflection coordinates was investigated by simulation, with the details described in Appendix *B* [App appb]. A Gaussian axisymmetric fibril orientation distribution with a mean tilt of 13° and standard deviation of 5° was used for the simulation. Gaussian errors with a standard deviation of one detector pixel spacing were added to the reflection coordinates, which were then used to calculate φ and β as described above. The resulting distributions of the estimated φ and β are shown in Appendix *B* [App appb]. The broader and long-tailed β distribution is evident in Fig. 15(*b*) in Appendix *B* [App appb]. Although the shapes of the simulated distributions are somewhat different to those observed, their overall characteristics match quite well, as described in Appendix *B* [App appb]. We conclude, therefore, that the broader and long-tailed distribution for the estimated β is due to the errors in measurement of the reflection positions, and that the distribution of the estimated φ better represents the true distributions.

### 4.4. Merging in two-dimensional reciprocal space   

Using the values of φ and β determined for each diffraction pattern, each pattern is mapped into two-dimensional reciprocal space 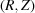. This is done by mapping each detector pixel to a position in reciprocal space and distributing the intensity over the four nearest-neighbour reciprocal-space grid points using bilinear interpolation. The mapped intensities are multiplied by the factor 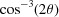 to account for the Jacobian between reciprocal space and detector space (Fraser *et al.*, 1976 ▸; Millane & Arnott, 1986 ▸; Millane, 2010 ▸). These patterns are then averaged together to give a merged two-dimensional diffraction pattern. An averaged background is calculated as described in Section 4.1, mapped to reciprocal space and subtracted from the merged frames. The resulting merged diffraction pattern is shown in Fig. 4 ▸(*c*).

Comparison of Figs. 4 ▸(*b*) and 4 ▸(*c*) shows a considerable reduction in the apparent disorientation. The merged pattern of Fig. 4 ▸(*c*) is the equivalent of a conventional fibre diffraction pattern in which the disorientation has been reduced, ideally, to zero, since the contributing pattern from each individual fibril has been mapped to the correct position in reciprocal space. Residual evidence of disorientation is due to errors in estimating φ, and particularly β, for the individual patterns. The reduction in the disorientation is illustrated in Fig. 5 ▸, which shows the angular profiles through the same equatorial and first layer line reflections (black and green curves) as used for the profiles from the averaged patterns in Fig. 4 ▸(*b*). The standard deviation of the intensity distribution has been reduced from 3° to 0.3° for the equatorial reflection. For the reflection on the first layer line, the standard deviation has also been considerably reduced, but is larger than for the equatorial reflection as a result of residual peak broadening in the *R* direction. Computational alignment of the diffraction patterns therefore gives a degree of alignment that would be very difficult, or impossible, to achieve with conventional fibre specimens. The improved alignment has the important effect of extending the resolution. Comparison of Figs. 4 ▸(*b*) and 4 ▸(*c*) shows that the resolution has been extended from about 3.6 Å to about 2.9 Å.

An important point to note is that the region around the meridian in the upper half of the pattern in Fig. 4 ▸(*c*) is well filled in compared with the same region in Fig. 4 ▸(*b*) because of the variety of tilts present in the specimen. This is because the averaging in Fig. 4 ▸(*b*) assumes, erroneously, that all fibrils have the same tilt, and the missing region around the meridian in Fig. 4 ▸(*b*) is a result of this single tilt value. However, Fig. 4 ▸(*c*) is assembled using the correct tilts of the individual fibrils, and since a range of tilts occur for the individual fibrils, the missing regions in reciprocal space for individual tilt values are filled in in the merged diffraction pattern. The result is that, if the range of tilts approaches the average tilt value, then reciprocal space is well sampled around the meridian to quite high resolution, as is evident in Fig. 4 ▸(*c*). This would be hard to achieve in conventional fibre diffraction experiments where one does not have access to the tilts of the individual mol­ecules, and could be approximated only by using multiple fibre specimens with a range of average tilts. The more complete access to reciprocal space achieved here therefore represents a further advantage of our method over conventional fibre diffraction analysis.

**Figure 8:**
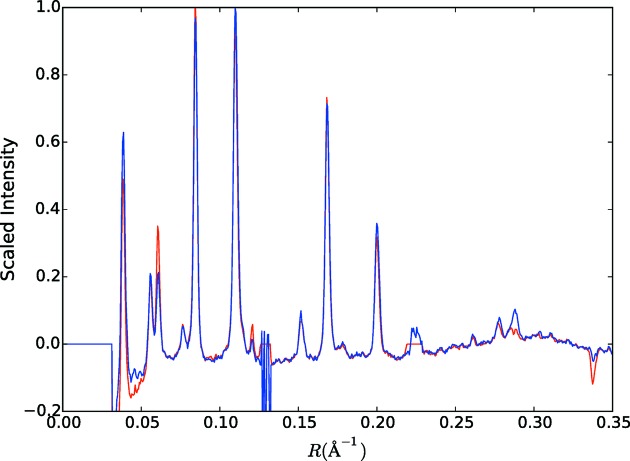
Intensity through the equator of Fig. 4 ▸(*b*) (red) and through the equator of Fig. 4 ▸(*c*) (blue), as described in the text.

To check that no bias is introduced by the selection of the patterns included in Fig. 4 ▸(*c*) (*i.e.* 33 788 of the 43 709 patterns), intensity profiles (calculated by averaging over a sector of angle ±5°) through the equator of Figs. 4 ▸(*b*) and 4 ▸(*c*) are compared in Fig. 8 ▸. Inspection of the figure shows that any differences are small, and the selection process does not appear to have introduced any systematic bias.

### 4.5. Cell constants   

The sharp reflections on the equator and the first layer line in Fig. 4 ▸(*c*) show that the molecules pack together in the lateral plane, forming small crystallites. The positions of the reflections allow the unit-cell constants to be determined. We assume that the crystallographic *c* axis is parallel to the orientation axis, and that the unit-cell angles, α and β (not to be confused with the fibre tilt angle), are 90°. Exceptions to this do occur with crystalline fibres, but they are quite rare. In this case, the *R* value of an equatorial reflection with Miller indices *h* and *k*, 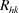, is given by 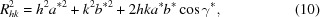
where 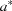, 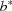 and 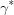 are reciprocal cell constants.

A Gaussian profile plus a linear background is fitted to each peak profile along the *R* direction, after averaging over 11 samples in *Z*. For peaks for which the standard deviation of the estimate of the centre of the Gaussian profile is less than one reciprocal-space grid spacing, the centre is used as an estimate of *R* for that reflection, denoted 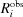. This gives 16 equatorial peak positions. The cell constants are determined by minimizing the difference between 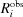 and 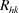 in equation (10) using weighted least-squares, with weights set to the inverse of the standard deviation of 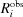. This gives cell constants *a* = 18.37 Å, *b* = 26.66 Å  and γ = 102.5°. Calculating the *R* values for all reciprocal-lattice points and comparing with the measured positions of all reflections gives a maximum difference between the measured and calculated 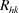 values of 0.003 Å^−1^. This value is less than the precision of the measurement of the peak positions which is about 0.005 Å^−1^, so that these cell constants are consistent with all the equatorial diffraction data. The measured and calculated *R* values are listed in Table S1 in the supporting information. Note that, as is usual with cylindrically averaged diffraction data, many of the reflections correspond to composite reciprocal-lattice points.

**Figure 9:**
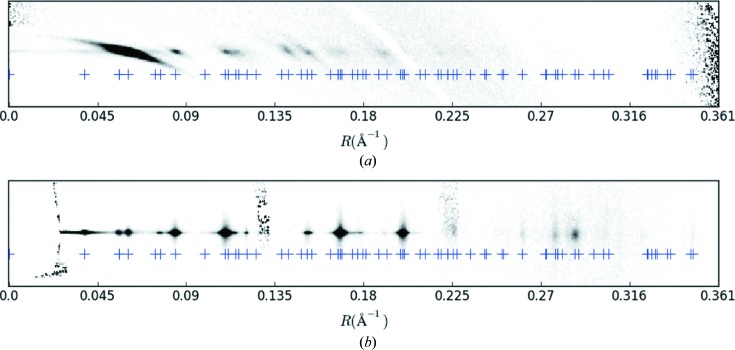
The equator (bottom) and first layer line (top) of the merged diffraction pattern (Fig. 4 ▸ *c*), and the cylindrically projected reciprocal-lattice points (+). Note that every diffraction spot is associated with at least one reciprocal-lattice point.

The *R* coordinates of the peaks on the first layer line are also listed in Table S1 and are seen to be consistent with the derived cell constants. The *Z* coordinates of the reflections on the first layer line were similarly measured and fitting to these spacings gives *c* = 4.82 Å. The positions of the cylindrically projected reciprocal-lattice points on the equator and first layer line are shown in Fig. 9 ▸. The derived cell constants are compared with those of related amyloid-like oligopeptide crystal structures in Appendix *C* [App appc].

### 4.6. Determination of ω   

Since diffraction data are available from individual fibrils, there is the possibility of orienting each pattern in three-dimensional reciprocal space and thus avoiding cylindrical averaging. To achieve this, the angle ω (which is defined here as the angle between 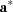 and the negative 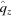 axis) must be determined for each diffraction pattern or each fibril. The cylindrically averaged data merged in two-dimensional reciprocal space as described above are still informative, and in particular they allow determination of the cell constants that aid in determining ω and assembling and merging the diffraction patterns in three-dimensional reciprocal space. Again, the difficulty is that of determining this angle for patterns that frequently contain only two reflections. We solve this problem by using the known φ and β angles for each pattern, coupled with knowledge of the cell constants, as described below.

The principle of the approach is as follows. For a particular diffraction pattern, for each observed reflection, the known *R* coordinate, together with the cell constants, gives a small number of possible indices 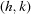 that are consistent with that *R* value. Since φ and β are known, there are only two values of ω that put each possible indexing assignment into a diffracting condition. There will then be a small number of possible ω values for each reflection. Since the single correct ω value is the same for all reflections on a single pattern, the values that are common over all the reflections are determined. There will frequently be only one such ω value. Patterns for which there is not a single consistent ω value are likely to be due to multiple fibrils and are discarded.

**Figure 10:**
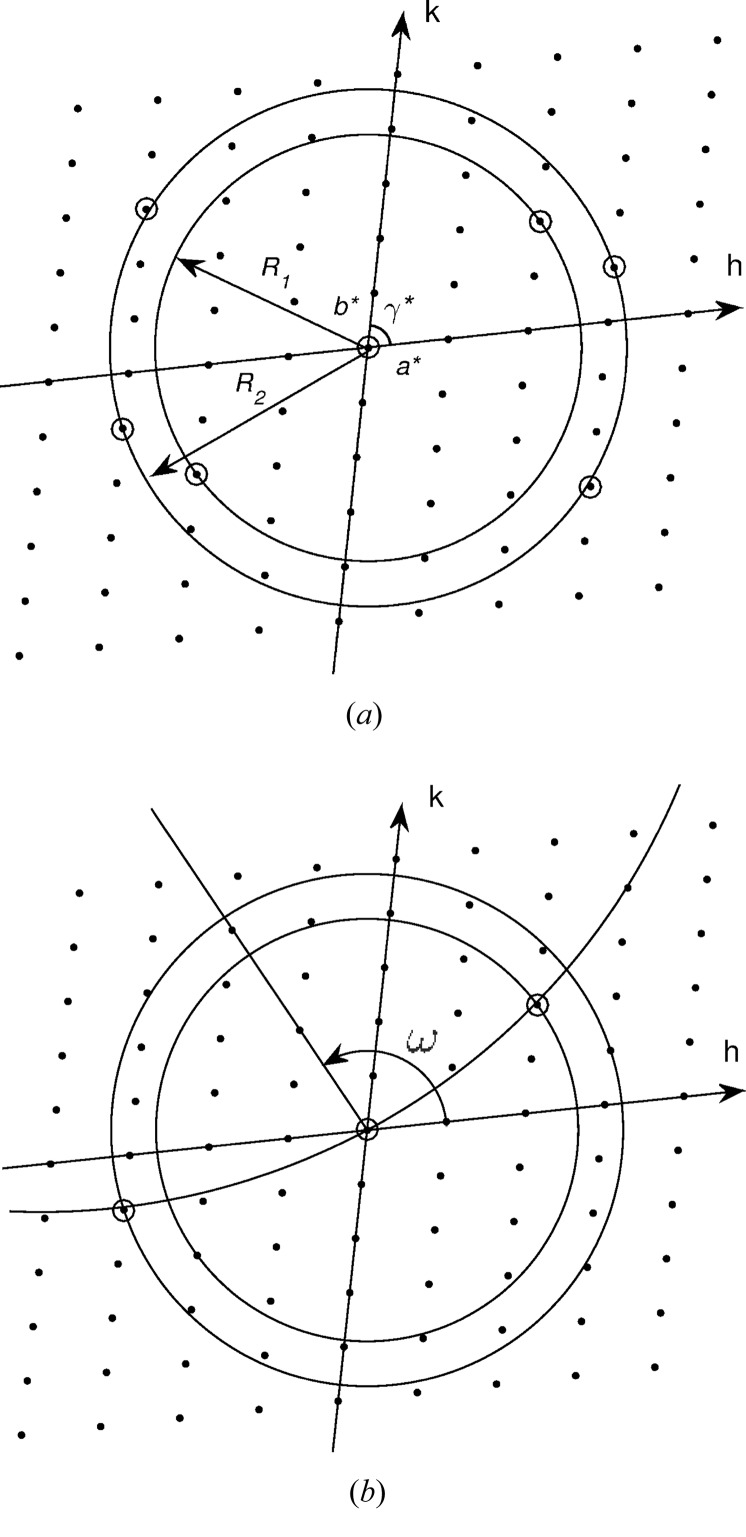
Illustration of the determination of ω for a pattern with two reflections. (*a*) The two observed peaks have radii *R*_1_ and *R*_2_ that correspond to one and two possible indexings (two and four indexings including their centrosymmetric mates) or reciprocal-lattice points, respectively. (*b*) Only one Ewald sphere section of radius cos(β)/λ (curved line) fits to one reciprocal-lattice point of radius *R*_1_ and one of radius *R*_2_, within a tolerance *T*, which defines a unique ω.

This approach is implemented as follows, and is illustrated in Fig. 10 ▸. Since the best-defined reflections in the current data set are on the equator, the analysis is based on the equatorial reflections only. All reflections on a single diffraction pattern are treated together, with all possible indexing combinations considered for each. For a pattern that has *N* peaks, with each peak *i* having 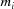 possible indexes, there are *P* = 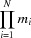 possible indexing combinations for the *N* peaks to consider. Note that often *N* = 2. The Ewald sphere intersects the *l* = 0 plane of the reciprocal lattice on a circle of radius cos(β)/λ that passes through the origin of reciprocal space. For a particular pattern, each observed peak is assigned a set of possible indices 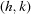 by requiring that 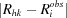 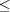 *T*, where *T* is a fixed tolerance (Fig. 10 ▸ *a*). For each of the *P* sets of reciprocal-lattice point assignments, a circle of radius cos(β)/λ passing through the origin is fitted to the *N* reciprocal-lattice points. The quality of fit is measured as the r.m.s. deviation from the circle to the reciprocal-lattice points. Acceptable indexings are those for which the circle passes within a distance *T* of each assigned reciprocal-lattice point. If there is a single acceptable indexing, then that indexing is assigned to the reflections and the value of ω is determined for that pattern (Fig. 10 ▸ *b*). If there are multiple acceptable indexings, then the ω corresponding to the best fit to the reciprocal-lattice points is assigned. Patterns for which there is no acceptable indexing are discarded.

There is information in addition to the *R* values for peaks on a diffraction pattern, since two peaks may occur on the same, or opposite, sides of the meridian. This further restricts the possible indexing assignments and ω values as follows. If peaks are on the same side of the meridian, then the assigned reciprocal-lattice points must be on the Ewald sphere section (circle) on the same side of the origin, *i.e.* reciprocal-lattice points on opposite sides of the origin are excluded. Likewise, if peaks are on opposite sides of the meridian, then the assigned reciprocal-lattice points must be on the Ewald sphere section on opposite sides of the origin. This further reduces the number of possible ω values.

The calculations described above were applied to the 33 788 frames used in the two-dimensional merge described in Section 4.3, using a tolerance *T* equal to the average peak width of the spots determined as described in Section 4.5 (which corresponds to 0.005 Å^−1^). Of these, unique ω values were assigned to 11 240 patterns.

### 4.7. Merging in three-dimensional reciprocal space   

With the angles 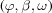 determined for each diffraction pattern, the patterns can be mapped onto three-dimensional reciprocal space and then merged together. Each detector pixel position is first mapped onto three-dimensional reciprocal space with φ = β = ω = 0, with resulting reciprocal-space coordinates denoted **X** = **q**. For each pattern, these positions are rotated by the corresponding angles φ, β and ω, about the 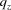, 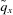 and 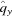 axes, respectively, to give their positions in reciprocal space, denoted 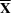, as 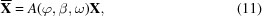
where 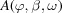 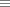 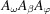 is the rotation matrix 
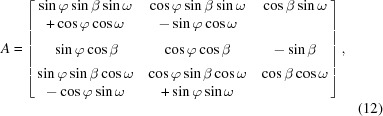
and 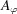, 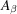 and 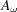 are the individual rotation matrices. The diffracted intensity at each detector pixel is then distributed over the neighbouring grid points in three-dimensional reciprocal space using trilinear interpolation.

**Figure 11:**
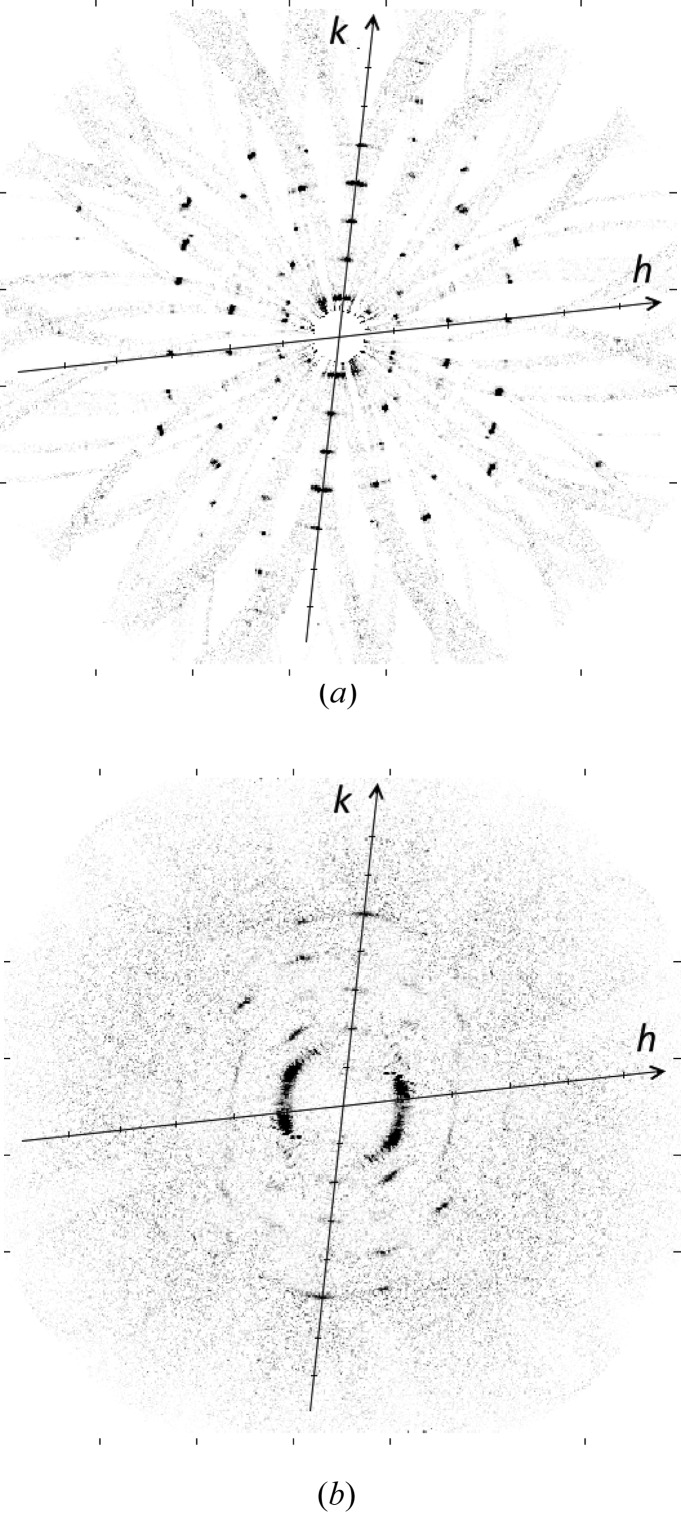
Three-dimensional merge of the diffraction data, (*a*) on the equatorial (*l* = 0) plane and (*b*) on the first (*l* = 1) layer plane, as described in the text.

The merge was conducted using the 11 240 patterns described in the previous section. A two-dimensional section through the equatorial plane (*l* = 0) of the intensity in reciprocal space, averaged over nine grid points normal to the plane, is shown in Fig. 11 ▸(*a*). The reciprocal lattice is clearly evident in the figure, with reflections seen out to ∼3.5 Å resolution.

Two features are evident in Fig. 11 ▸(*a*). First, there are faint broad arcs that pass through each reciprocal-lattice point and the origin of reciprocal space. These correspond to the discrete values of ω that give diffraction at a particular reciprocal-lattice point, and the diffraction for a particular ω lies along one of these arcs (each of which is an Ewald sphere section). The arcs are due to the more or less continuous background diffraction in the contributing diffraction patterns. This background is actually very weak but is shown in Fig. 11 ▸(*a*) since the contrast is artificially increased to show the weak reflections. The breadth of the arcs is due to the imprecise determination of the geometric parameters and the allowed tolerances in fitting each Ewald sphere section.

Second, inspection of Fig. 11 ▸(*a*) shows that at each reciprocal-lattice point there is not a single peak but a small number of sharp peaks, each offset slightly from each other. Each of these peaks represents diffraction at that reciprocal-lattice point, and the offset from the reciprocal-lattice point is due to the restricted number of fibril tilts, often none of which correspond exactly to a diffracting condition at that reciprocal-lattice point. Each sharp peak is due to a different reciprocal-lattice point that is paired with that point, and thus a different ω value, as described in Section 4.6. This phenomenon is described in detail in Appendix *D* [App appd].

A two-dimensional section through the first layer plane (*l* = 1) of the intensities in reciprocal space, averaged over nine grid points normal to the plane, is shown in Fig. 11 ▸(*b*). Overall, the intensity on this section is more diffuse, and with a higher background, than that on the equatorial plane. This is due to a number of factors, including the more diffuse and arced reflections (due in part to their more tangential intersection with the Ewald sphere), the higher background on the first layer line of the original diffraction patterns, and the fact that the orientation angles are determined from the equatorial data.

**Figure 12:**
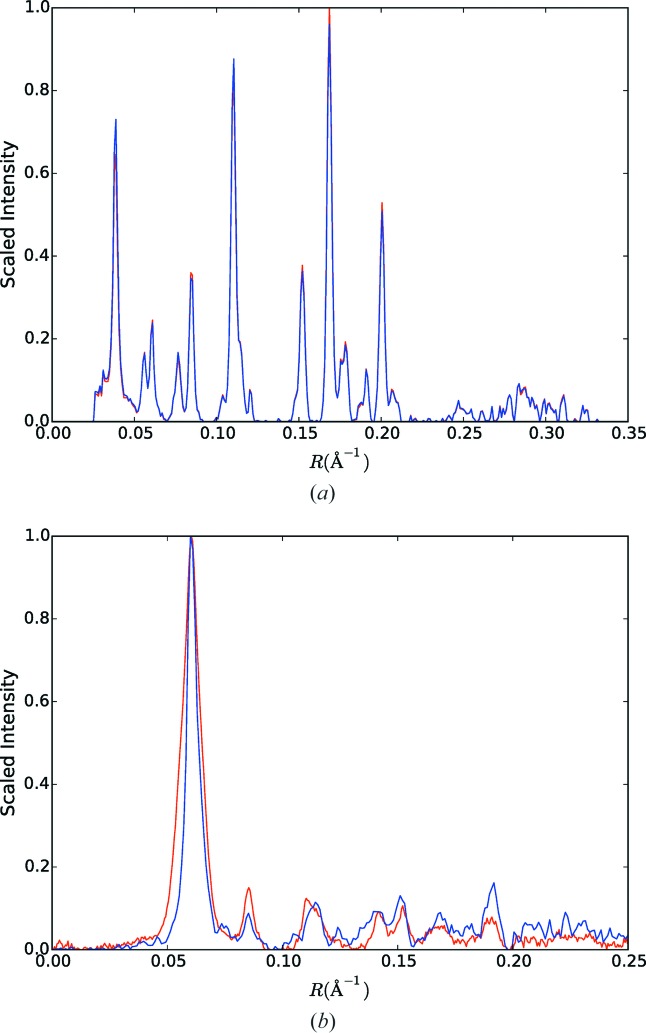
(*a*) Intensity profiles through the equator of the two-dimensional merged data (red) and of the cylindrically re-projected intensity through the equator of the three-dimensional merge (blue), as described in the text. (*b*) The same as panel (*a*) except for the *l* = 1 layer plane.

To check that no bias is introduced by the selection of patterns included in the three-dimensional merge, intensity profiles through the equator and first layer line of the two-dimensional merge and the cylindrically re-projected three-dimensional merge on the corresponding layer plane were calculated and are shown in Fig. 12 ▸. Inspection of the figure shows that any differences are small, and the selection process does not appear to have introduced any systematic bias.

### 4.8. Calculation of structure amplitudes   

**Figure 13:**
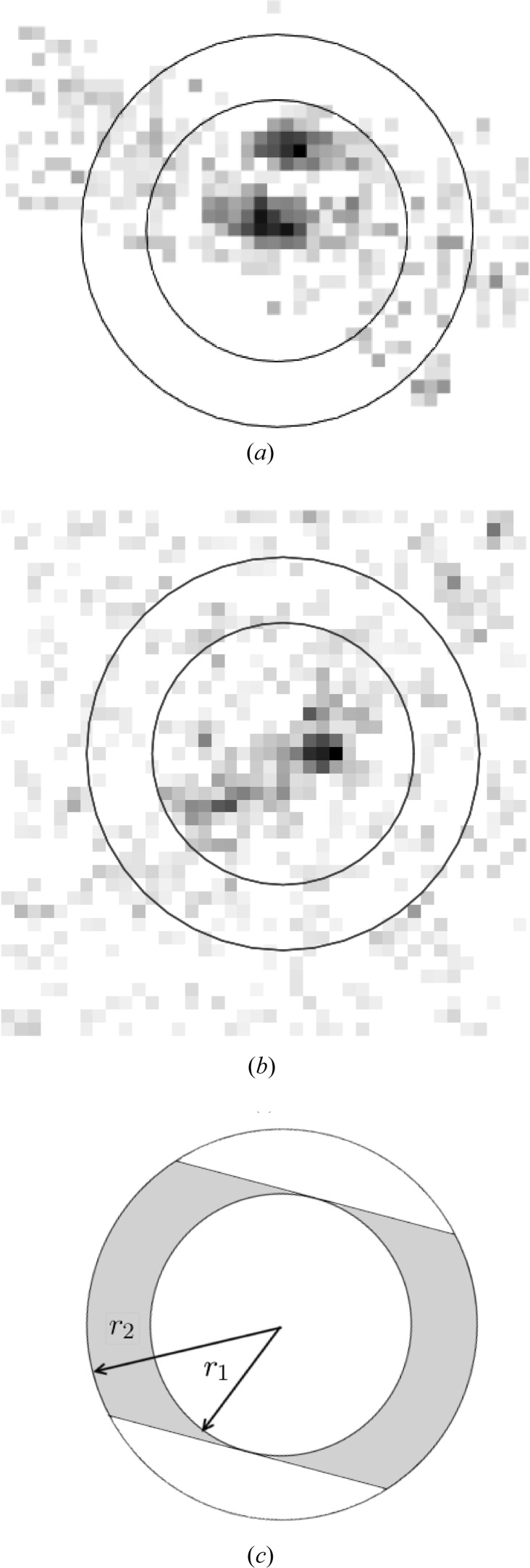
Diffracted intensity in a region around a reciprocal-lattice point in the three-dimensional merge and the integration cylinders, (*a*) on the equatorial and (*b*) on the first layer planes. (*c*) The region (shaded) where the background is measured.

The structure amplitudes are calculated by integrating the intensity in a region around each reciprocal-lattice point in three-dimensional reciprocal space. An example of the diffracted intensity around a reciprocal-lattice point in the equatorial plane is shown in Fig. 13 ▸(*a*). This shows two sharp peaks and the band of background intensity as described in the previous section. The background is low as a result of the background subtracted from the original diffraction patterns. The structure amplitude is estimated by integrating the intensity within a cylindrical region that encloses the contributing peaks, as shown in Fig. 13 ▸(*a*). The radius of the cylindrical region, denoted 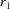, is expressed as a fraction of the smallest reciprocal-lattice spacing in the equatorial plane. The length of the cylinder, denoted 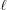, is expressed as a fraction of 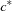.

Residual background is estimated using the intensity in a cylindrical shell surrounding the integration cylinder, with outer radius denoted 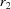 and length 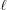 (Fig. 13 ▸). As described in the previous section, Ewald sphere sections that pass through the origin and the reciprocal-lattice point contribute most of the background. Since the radius of the Ewald sphere is large, the background is estimated using the intensity in a band of width 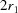 within the cylindrical shell, as shown in Fig. 13 ▸(*c*). This band occupies a fraction *f* of the cylindrical shell given by 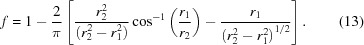
The average background per pixel contributing to the integrated intensity is therefore calculated as the average value of the fraction *f* of the largest intensities in the cylindrical shell. The corresponding background value is subtracted from the integrated intensity to estimate the structure amplitude.

Inspection of the extent of the sharp peaks contributing to each reflection indicates suitable values 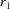 = 0.25, 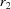 = 0.38 and 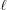 = 0.03, which correspond to grid spacings of 10, 15 and 10, respectively, in the case here. The structure amplitudes on the equatorial plane are calculated as described above using these values. The estimated structure amplitudes are listed in Table S2 in the supporting information. Amplitudes that are calculated as negative are listed as below the threshold in Table S2. The structure amplitudes 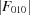, 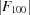 and 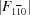 are unreliable due to proximity to the mask and to their extended volume, as seen in Fig. 11 ▸(*a*).

**Figure 14:**
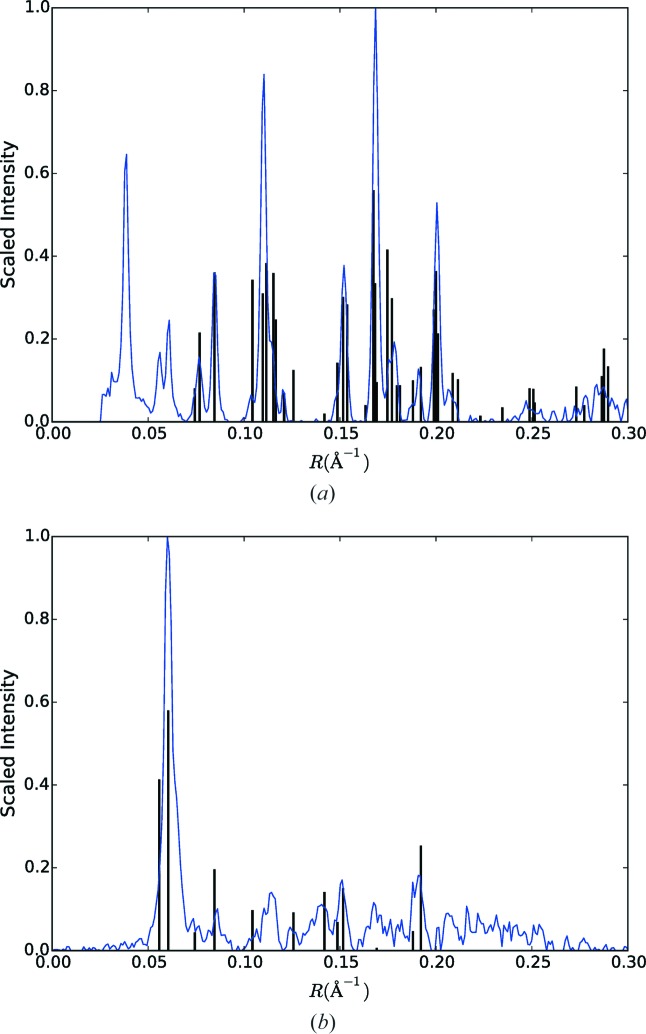
Cylindrically averaged intensity profiles (blue curve) overlaid with the measured structure intensities (bars) for (*a*) *l* = 0 and (*b*) *l* = 1.

**Figure 15:**
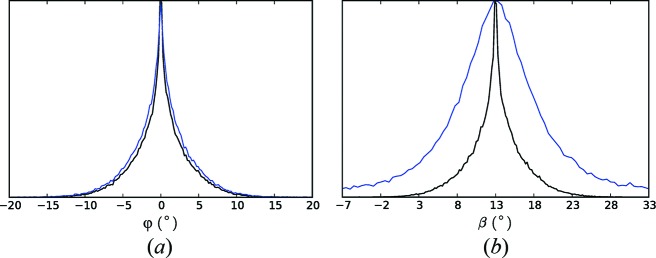
Simulation of the determination of φ and β. (*a*) Distributions of the true values of φ (black) and the estimated values 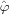 (blue). (*b*) Distributions of the true values of β (black) and the estimated values 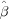 (blue).

On the *l* = 1 layer plane, as described above, the reflections and the background are more diffuse. The intensity around a reciprocal-lattice point on the *l* = 1 plane is shown in Fig. 13 ▸(*b*). The structure amplitudes are calculated in the same way using the same size integration regions, and are also listed in Table S2. Note that, since the angles ω and ω + 180° cannot be distinguished, and also since the fibrils will be randomly ‘up’ and ‘down’ in the jet, the reflections 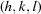 and 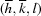 cannot be distinguished. Therefore, the structure amplitudes 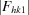 listed in Table S2 actually represent the quantity 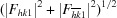. We did not attempt to estimate the value of the strong 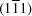 reflection. The squared structure amplitudes overlayed with the cylindrically averaged diffraction intensity on the equator and first layer lines are shown in Fig. 14 ▸.

To check the consistency of the derived structure amplitudes, the original patterns were divided into two random sets, a three-dimensional merge constructed for each and two sets of structure amplitudes calculated. Calculating the correlation coefficient and 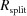 between the two sets of structure amplitudes gave values of 0.99 and 0.08, respectively, for the *l* = 0 data, and 0.97 and 0.13 for the full data set (*l* = 0 and *l* = 1).

## 5. Discussion   

We have collected serial X-ray diffraction data from single crystalline amyloid fibrils from the adenovirus shaft, using an X-ray free-electron laser. The fibrils delivered with a liquid injector are oriented within about 5° of a common axis. A large number of diffraction patterns were collected, about 10% of which contain detectable diffraction from the fibrils, and about 10% of these are due to a single fibril in the beam focus and were used for analysis. Most of these patterns show two equatorial peaks but, despite this small number, the orientation of the fibril long axes in the beam can be determined for many of the patterns. Using these orientations, each pattern is placed on a common axis in reciprocal space, and averaging of these patterns gives the equivalent of a conventional cylindrically averaged fibre diffraction pattern, but the computational alignment reduces the disorientation to less than 1° and increases the resolution of the data. The cylindrically averaged pattern is used to determine the cell constants. Using the reciprocal-lattice information allows the rotation of individual fibrils about their fibril axis to be determined for about 30% of the patterns, and the patterns to be correctly positioned in three-dimensional reciprocal space. Averaging of these patterns then gives a picture of the three-dimensional diffraction by the fibril, allowing the individual structure amplitudes to be measured. The result is a set of three-dimensional structure amplitudes that can be used for structure determination as in conventional single-crystal crystallography, by, for example, molecular replacement. Such an analysis is underway for the adenovirus amyloid using the data described here. This general procedure overcomes the problems of orientation and cylindrical averaging that plague conventional fibre diffraction analysis (which allows measurement of only a two-dimensional diffraction data set).

The method explored here has potentially wide application in structural studies of the many biological systems that form rod-like assemblies. Such systems are ubiquitous in biology and perform a wide variety of important functions. Their tendency to aggregate in partially aligned and partially disordered macroscopic specimens with random rotations places severe limitations on structural studies using such specimens. The opportunity to measure diffraction from individual assemblies using new high-intensity/short pulse-duration X-ray sources opens up new opportunities to study the structures of such systems that avoid the problems associated with disordered macroscopic fibrous aggregates. The utility of the method will depend on the development of suitable specimen preparation methods for other fibrilar biological systems.

The methods described here utilize crystalline fibrils that have advantages in terms of signal level and ease of orientation determination. However, it is likely that many aspects of the approach described here can be utilized in developing related methods for the analysis of data from noncrystalline fibrils. The use of noncrystalline fibres has the disadvantage that the scattering is weaker, but the advantage that the phase problem is better constrained and *ab initio* phasing may be feasible (Millane, 2017 ▸).

There are a number of avenues through which this method could be improved. The ultimate hit rate is low (∼0.3%) as a result of the weakly scattering fibrils and the necessity of using a low concentration to increase the proportion of patterns due to a single fibril. However, this problem will be eased by the increased flux and higher pulse repetition rates that will be available at new XFELs such as the European XFEL. We used only the equatorial data to determine fibril orientations as a result of the better precision of this data and the simpler analysis. We did attempt to re-refine the tilt during the fitting of patterns to the reciprocal lattice, although only small improvements were seen. However, investigation of the use of off-equatorial data and co-refinement of cell constants and fibril orientations would be fruitful. An expand–maximize–compress (EMC) approach to orientation determination (Loh & Elser, 2009 ▸) is potentially feasible, although the advantage of such an approach is not clear in the case of the relatively strong sampled (Bragg) data in patterns from crystalline fibrils, as opposed to the weak patterns characteristic of single-particle imaging. We note that Popp *et al.* (2017 ▸) used EMC for reorientation of some of their patterns from noncrystalline filaments. In the work reported here, we have averaged the full diffraction patterns in three-dimensional reciprocal space and measured the structure amplitudes from the merged data set. An alternative approach is to measure (noisy) structure amplitudes in each pattern and average these, as is generally done in SFX data processing (Kirian *et al.*, 2010 ▸; White *et al.*, 2012 ▸). In this scenario, since the full patterns are not merged, there may be savings in terms of computational cost.

The methods described here could, in principle, be applied to poorly oriented, or even completely disorientated, fibrils that are not easily aligned. However, there will be additional difficulties due to the wide variation in φ and β values which will complicate initial interpretation of the single-shot diffraction patterns. Suitable methods for analysis of such patterns with only a few reflections would be needed.

## Supplementary Material

Additional tables. DOI: 10.1107/S2052252517014324/sp5001sup1.pdf

## Figures and Tables

**Figure 16:**
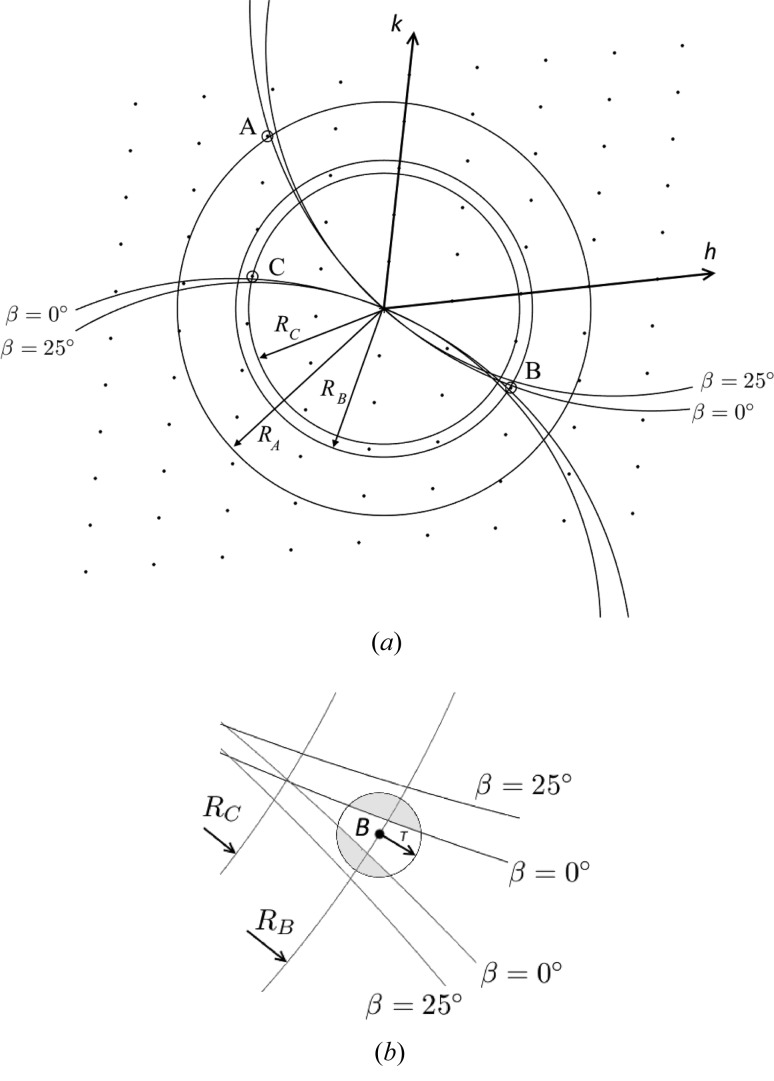
Illustration of fitting Ewald sphere sections, of radius cos(β)/λ, to pairs of reciprocal-lattice points. (*a*) Three observed peaks with radii 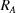, 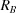 and 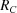, and specimen tilts between 0 and 25°, are considered. The best indexings for β = 0° and β = 25° are shown. The indexing of both pairs of radii share the common reciprocal lattice point *B*. (*b*) A close-up around reciprocal lattice point *B*. In both indexing cases, the Ewald sphere never intercepts point *B* for the range of β values present (0°, 25°). Moreover, indexing is not possible for β larger than particular values as the Ewald sphere is not within the accepted tolerance radius *T* of the reciprocal lattice point *B*. As a result, only the regions shaded grey can accrue diffraction for the two indexing scenarios, leading to a fragmented intensity distribution about the reciprocal lattice point *B* in the merged data.

**Table 1:** Cell constants of the adenovirus octapeptide and two other crystal structures (Nelson *et al.*, 2005 ▸)

	Adenovirus	NNQQNY	GNNQQNY
*a* (Å)	18.37	21.15	21.94
*b* (Å)	26.66	23.13	23.48
*c* (Å)	4.82	4.87	4.87
γ (°)	102.5	102.93	107.08
Density (*D* Å^−3^)	0.347	0.335	0.348

## Appendix A Peak broadening and fibril dimensions

The reflections in Fig. 4 ▸ are broadened as a result of fibril dimensions, possible disorder, disorientation and instrumental effects. Estimates of fibril dimensions can potentially be obtained from measurements of peak broadening, as long as instrumental effects can be accounted for. The effect of disorientation can be circumvented by considering the widths of the equatorial reflections in the *R* direction and the widths of the near-meridional peaks in the *Z* direction. Aside from specimen effects, the other primary effect in the case here is from beam broadening. The horizontal and vertical beam divergence (FWHM) are 1 and 2 mrad, respectively, and for this experimental geometry they correspond to a broadening of FWHM_H_ ≃ 0.0005 Å^−1^ and FWHM_V_ ≃ 0.0010 Å^−1^ at the detector.

Measurement of the FWHM of the equatorial peaks in the *R* direction gives FWHM_*R*_ = 0.0030 Å^−1^. The broadening due to the specimen in the lateral direction, denoted FWHM_*x*_, is related to the above quantities by  +  ≃ , which gives  ≃ 0.0030 Å^−1^. The mean width  of the diffracting particles, estimated by the Scherrer equation in the form

gives a mean fibril width of about 300 Å. Estimation of fibril widths from electron micrographs of the specimen (Fig. 1 ▸) gives a value of about 200 Å, which, given the uncertainties in the above quantities, is consistent with that from the X-ray data.

Measurement of the FWHM in the *Z* direction of reflections on the first layer line close to the meridian gives a value FWHM_*Z*_ ≃ 0.0030 Å^−1^. Using the value of FWHM_V_ above and making a similar calculation to that above gives an axial FWHM in the *z* direction due to the specimen FWHM_*z*_ ≃ 0.0028 Å^−1^, which gives a coherence length of the fibrils  of about 320 Å. The fibril lengths seen in the electron micrographs vary between about 1000 and 4000 Å (Fig. 1 ▸), although the X-ray focal spot size of ∼2000 Å will result in an apparent shortening of the longer fibrils. The coherence length from the X-ray data is therefore smaller than the average fibril length seen in micrographs, within the caveat of the precision of the above quantities.

Given the likely structure of the fibrils as a stack of a large number of hydrogen-bonded β-strands, cumulative (paracrystalline) disorder in the stacking distance is a possible source of reduced coherence length. The FWHM of diffraction peaks due to paracrystalline disorder (ignoring finite length effects, which will be small here due to the long filaments) is given by (Guinier, 1963 ▸; Eads & Millane, 2001 ▸)

where σ is the standard deviation of the interstack spacing and *n* is the order of the reflection. Substitution into equation (15) (*n* = 1 and *c* = 4.82 Å) shows that a standard deviation in the interstack spacing of 0.05 Å is sufficient to produce the observed FWHM_*z*_ of 0.0028 Å^−1^ at the first layer line. Such a variation in the length of the hydrogen bonds that stabilize adjacent β-strands is well within reason.

## Appendix B Simulation of errors in the estimated φ and β

The effect of errors in the measured equatorial reflection positions on the estimated values of φ and β is examined by simulation. Fibril orientations are assumed to be axisymmetric with respect to the mean orientation axis. The angle α between a fibril and the mean orientation axis is modelled as zero-mean, normally distributed with a standard deviation α_0_. The tilt of the mean orientation axis is β_0_. For each fibril, the azimuth around the mean orientation axis is selected from a uniform distribution, and the corresponding angles φ and β are calculated. Two equatorial reflection *x* coordinates on the detector are selected from a normal distribution with means  and standard deviation σ_*x*_. Using equations (1) and (8), the following quadratic equation for the *y* coordinate of a reflection is derived:

where

The *y* coordinates of the two reflections are calculated by solving equation (16). Normally distributed zero-mean errors, with standard deviation σ, are added to the *x* and *y* coordinates of each reflection. The φ and β values are then calculated from these two reflection coordinates using equations (8) and (9).

For our simulations, we used the values α_0_ = 5°, β_0_ = 13°, σ = 1 px, *x*_0_ = 400 px and σ_*x*_ = 200 px, and the calculation was repeated for 10^5^ fibrils. The distributions of the true angles φ and β, and their estimated values  and , are shown in Fig. 15 ▸. Inspection of the figure shows that, while the distribution of the estimated φ values matches the true distribution reasonably well, the distribution of the estimated β is broader and longer tailed than the true distribution. The FWHMs of the simulated estimated φ and β are 2.6° and 10.3°, respectively, which compare reasonably well with the measured values of 5.6° and 10.0° described in Section 4.3.

## Appendix C Comparison of cell constants

The overall features of the diffraction pattern indicate that the octapeptide molecules form filaments of stacked β-sheets, although the structural details are not yet defined. The absent or weak diffraction near the meridian on the first layer line indicates at least approximate 2_1_ screw symmetry along the long axis. The crystal structures of two amyloid-like hexa- and hepta-peptide molecules have been reported by Nelson *et al.* (2005 ▸), and it is therefore interesting to compare the cell constants obtained here with those of these two structures. The three sets of cell constants are listed in Table 1 ▸. Inspection of the table shows that, although the cell constants are different, there are overall similarities that indicate that the adenovirus shaft amyloid molecule may pack overall in a similar manner to the other two molecules. This is supported by the similar packing densities. The one longer and one shorter cell axis of the adenovirus cell compared with the other two sequences may be due to the longer sequence but fewer side chains of the former compared with the latter. In summary, the cell constants determined here for the amyloid-forming adenovirus octapeptide are consistent with what might be expected from those of similar size amyloid-like sequences, indicating an overall similar kind of packing.

## Appendix D Fitting Ewald spheres to the reciprocal lattice

Merging in three-dimensional reciprocal space and the presence of multiple peaks associated with a reciprocal lattice point is illustrated in Fig. 16 ▸.
