# Supplementary material for: Analysis of XFEL serial diffraction data from individual crystalline fibrils
Source: IUCrJ. 2017 Oct 20;4(Pt 6):795–811. doi: 10.1107/S2052252517014324 (PMC5668865; doi:10.1107/S2052252517014324)
Supplement: Supplementary file 1 [file m-04-00795-sup1.pdf]

Analysis of XFEL serial diffraction data from individual crystalline fibrils

David H. Wojtas ,[a] Kartik Ayyer ,[b] Mengning Liang ,[c] Estelle Mossou ,[d,i] Filippo Romoli ,[k] Carolin Seuring ,[b] Kenneth R. Beyerlein ,[b] Richard J. Bean ,[g] Andrew J. Morgan ,[b] Dominik Oberthuer ,[b] Holger Fleckenstein ,[b] Michael Heymann ,[b,h] Cornelius Gati ,[b] Oleksandr Yefanov ,[b] Miriam Barthelmess ,[b] Eirini Ornithopoulou ,[j] Lorenzo Galli ,[b] P. Lourdu Xavier ,[b,m] W. L. Ling ,[n] Matthias Frank ,[l] Chun Hong Yoon ,[c] Thomas A. White ,[b] Saša Bajt ,[p] Anna Mitraki ,[j] Sebastien Boutet ,[c] Andrew Aquila ,[c] Anton Barty ,[b] V. Trevor Forsyth ,[i,d] Henry N. Chapman [b,e,f] and Rick P. Millane [a*]

[a]*Department of Electrical and Computer Engineering, University of Canterbury, Christchurch, New Zealand,* [b]*Center for Free-Electron Laser Science, DESY, Hamburg, Germany,* [c]*Linac Coherent Light Source, SLAC National Accelerator Laboratory, Menlo Park, California, USA,* [d]*Institut Laue-Langevin, Grenoble, France,* [e]*Department of Physics, University of Hamburg, Hamburg, Germany,* [f]*Centre for Ultrafast Imaging, University of Hamburg, Hamburg, Germany,* [g]*European XFEL GmbH, Hamburg, Germany,* [h]*Max Planck Institute of Biochemistry, Martinsried, Germany,* [i]*Faculty of Natural Sciences, Keele University, UK,* [j]*Department of Materials Science and Technology, University of Crete and IESL/FORTH, Crete, Greece,* [k]*European Synchrotron Radiation Facility, Grenoble, France,* [l]*Lawrence Livermore National Laboratory, Livermore, California, USA,* [m]*Max-Planck Institute for the Structure and Dynamics of Matter, Hamburg,*

Germany, [n] *Université Grenoble Alpes, Grenoble, France, and* [p] *Photon Science, DESY, Hamburg, Germany. E-mail: rick.millane@canterbury.ac.nz*

1. Supplementary information

Table 1. *Measured and predicted R values.*

(h,k)	R_{hk}(Å^{-1})	R_i^{obs}(Å^{-1})	ΔR(Å^{-1})	Intensity	R_i(Å^{-1})	ΔR(Å^{-1})	Intensity
(0,1)	0.0385	0.0387	0.0002	?			
(1,0)	0.0559	0.0557	-0.0002	m	0.0589	0.003	vs
(-1,1)	0.0604	0.0605	0.0001	m		-0.0015	
(1,1)	0.0745	0.0764	0.0019	w	0.0743	-0.0002	w
(0,2)	0.077		-0.0006			-0.0027	
(-1,2)	0.0845	0.0843	-0.0002	s	0.0843	-0.0002	m
(1,2)	0.1047						
(-2,1)	0.1098	0.1098	0.0000	s	0.1103	0.0005	m
(2,0)	0.1117		-0.0019			-0.0014	
(0,3)	0.1155				0.1156	0.0001	w
(-1,3)	0.1166					-0.001	
(-2,2)	0.1208	0.1207	-0.0001	w			
(2,1)	0.126						
(1,3)	0.139				0.1416	0.0026	w
(-2,3)	0.1417					-0.0001	
(2,2)	0.1491	0.1512	0.0021	m		0.0026	
(-1,4)	0.1517		-0.0005		0.1517	0	m
(0,4)	0.1539					-0.0022	
(-3,1)	0.1634						
(3,0)	0.1676	0.1682	0.0006	s	0.1676	0	w
(-3,2)	0.1682		0.0000			-0.0006	
(-2,4)	0.1689		-0.0007			-0.0013	
(1,4)	0.175						
(2,3)	0.1776	0.1782	0.0006	vw			
(3,1)	0.1801		-0.0019				
(-3,3)	0.1812		-0.0030				
(-1,5)	0.1881						
(0,5)	0.1924						
(-2,5)	0.1993	0.2	0.0003	s	0.1989	-0.0004	vw
(3,2)	0.1999		0.0001			-0.001	
(-3,4)	0.2008		-0.0007			-0.0019	
(2,4)	0.2093						
(1,5)	0.2119						
(-4,1)	0.2181						
(-4,2)	0.2196						
(4,0)	0.2234						
(3,3)	0.2236						
(-3,5)	0.2254						
(-4,3)	0.2276						
(4,1)	0.235						
(-4,4)	0.2416						
(2,5)	0.243						
(3,4)	0.2515						
(4,2)	0.252						
(-4,5)	0.2605	0.261	0.0005	vw			
(-5,2)	0.2727						
(-5,1)	0.2733						
(4,3)	0.2733						
(-5,3)	0.2775	0.2774	-0.0001	w			
(5,0)	0.2793		-0.0019				
(3,5)	0.2818						
(-5,4)	0.2874	0.2875	0.0001	m			
(5,1)	0.2903		-0.0028				

R_{hk} are values for the derived unit cell and R_i^{obs} are the measured values. $\Delta R = R_i^{obs} - R_{hk}$. Peak intensities are denoted: vw = very weak, w = weak, m = medium, s = strong, vs = very strong.

Table 2. *Measured structure amplitudes* $|F_{hkl}|$.

(h,k)	R_{hk} (Å^{-1})	F_{hk0}	F_{hk1}
(0 ,0)	0.0	U	T
(0 ,1)	0.0384	U	T
(0 ,2)	0.0769	38	T
(0 ,3)	0.1153	64	T
(0 ,4)	0.1537	51	T
(0 ,5)	0.1922	24	33
(0 ,6)	0.2306	T	-
(0 ,7)	0.2691	T	-
(1 ,-7)	0.2626	T	-
(1 ,-6)	0.2251	T	-
(1 ,-5)	0.1881	18	06
(1 ,-4)	0.1517	54	19
(1 ,-3)	0.1166	44	T
(1 ,-2)	0.0846	65	25
(1 ,-1)	0.0604	U	U
(1 ,0)	0.0558	U	54
(1 ,1)	0.0743	14	06
(1 ,2)	0.1044	61	13
(1 ,3)	0.1386	T	T
(1 ,4)	0.1746	74	T
(1 ,5)	0.2115	18	T
(1 ,6)	0.2488	14	
(1 ,7)	0.2865	20	
(2 ,-7)	0.2678	T	
(2 ,-6)	0.2332	T	
(2 ,-5)	0.2	65	T
(2 ,-4)	0.1691	17	01
(2 ,-3)	0.1419	03	18
(2 ,-2)	0.1209	12	T
(2 ,-1)	0.1098	55	T
(2 ,0)	0.1116	68	T
(2 ,1)	0.1257	22	12
(2 ,2)	0.1487	25	09
(2 ,3)	0.1771	53	T
(2 ,4)	0.2087	21	T
(2 ,5)	0.2424	T	
(2 ,6)	0.2772	07	
(3 ,-7)	0.2841	T	
(3 ,-6)	0.2537	T	
(3 ,-5)	0.2256	T	
(3 ,-4)	0.201	38	T
(3 ,-3)	0.1813	16	T
(3 ,-2)	0.1682	60	T
(3 ,-1)	0.1633	07	T
(3 ,0)	0.1673	100	T
(3 ,1)	0.1797	16	T
(3 ,2)	0.1988	48	T
(3 ,3)	0.223	T	
(3 ,4)	0.2508	14	
(3 ,5)	0.281	T	
(4 ,-6)	0.2837	T	
(4 ,-5)	0.2608	T	
(4 ,-4)	0.2418	T	
(4 ,-3)	0.2277	T	
(4 ,-2)	0.2196	T	
(4 ,-1)	0.218	T	
(4 ,0)	0.2231	02	
(4 ,1)	0.2345	06	
(4 ,2)	0.2514	08	
(4 ,3)	0.2726	T	
(4 ,4)	0.2973	T	
(5 ,-4)	0.2876	31	
(5 ,-3)	0.2776	T	
(5 ,-2)	0.2727	T	
(5 ,-1)	0.2731	15	
(5 ,0)	0.2789	T	
(5 ,1)	0.2897	24	

Unmeasured and below threshold values are denoted U and T, respectively. For $l = 1$, the values $|F_{hk1}|$ represent $\sqrt{|F_{hk1}|^2 + |F_{\bar{h}\bar{k}1}|^2}$, as described in the text.
